# Supplementary material for: The interactome of intact mitochondria by cross-linking mass spectrometry provides evidence for coexisting respiratory supercomplexes
Source: Mol Cell Proteomics. 2017 Dec 8;17(2):216–32. doi: 10.1074/mcp.RA117.000470 (PMC5795388; doi:10.1074/mcp.RA117.000470)
Supplement: Supplemental Data [file supp_RA117.000470_133922_0_supp_23978_fzffwf.zip › spectra_annotation/mito_spectra_annotation/178-1-3-1-11-1.pdf]

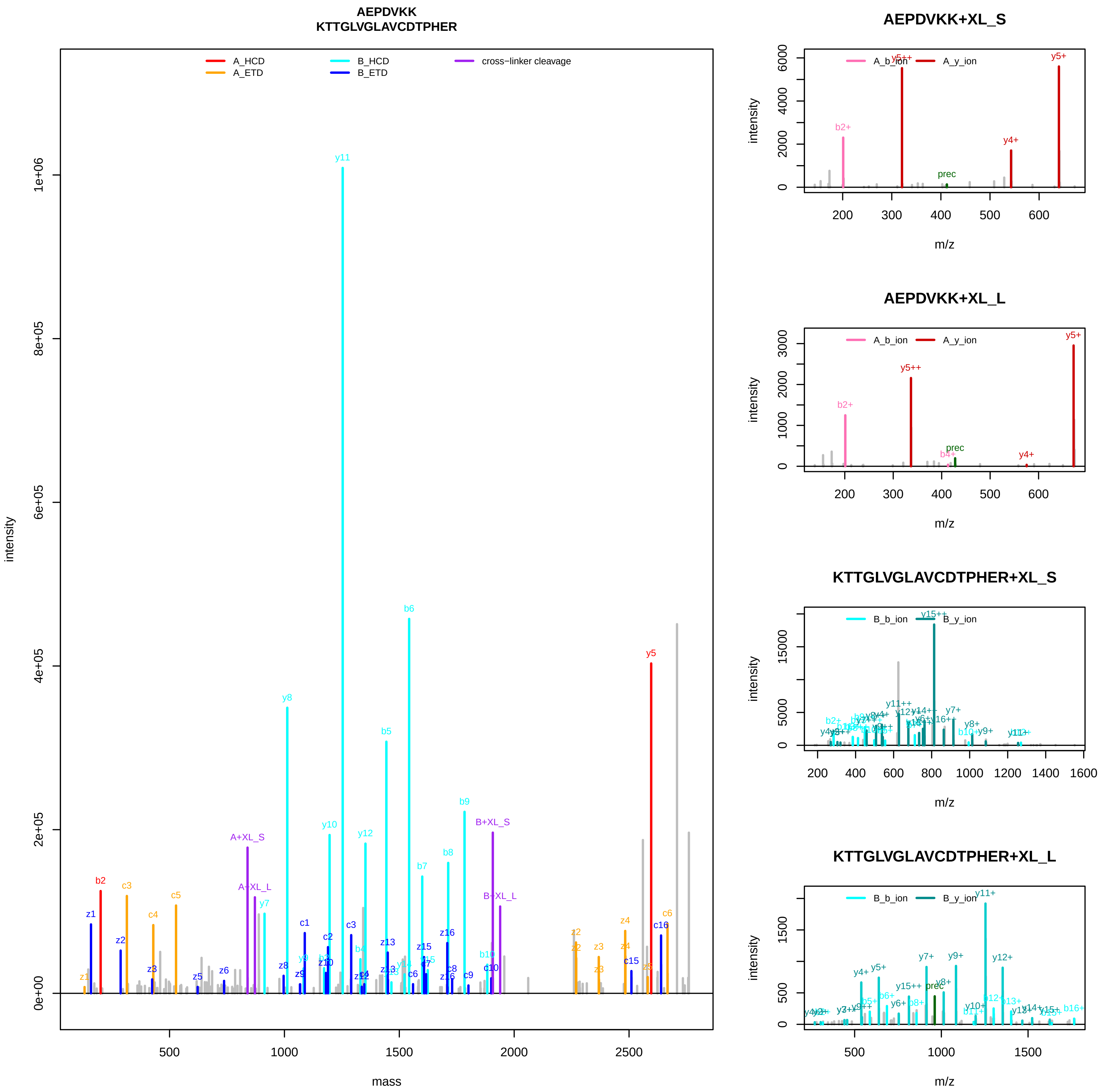
AEPDVKK
KTTGLVGLAVCDTPHER
A_HCD
A_ETD
B_HCD
B_ETD
cross−linker cleavage
intensity
mass
0e+00
2e+05
4e+05
6e+05
8e+05
1e+06
500
1000
1500
2000
2500
y11
b6
y5
y8
b5
b9
B+XL_S
A+XL_S
A+XL_L
B+XL_L
y10
y12
b8
b7
y7
b2
c3
c4
c5
c6
c1
c2
z1
z2
z3
z5
z6
z8
z9
z13
z15
z16
c15
c16
z4
AEPDVKK+XL_S
AEPDVKK+XL_L
KTTGLVGLAVCDTPHER+XL_S
KTTGLVGLAVCDTPHER+XL_L
A_b_ion
A_y_ion
B_b_ion
B_y_ion
m/z
b2+
y5++
y5+
y4+
b4+
prec
y15++
y11++
y12++
y14++
y7+
y8+
y9+
y11+
y4+
y5+
y6+
y12+
y15+
b16+
